# Effects of Stevia Extract on Postprandial Glucose Response, Satiety and Energy Intake: A Three-Arm Crossover Trial

**DOI:** 10.3390/nu11123036

**Published:** 2019-12-12

**Authors:** Grace Farhat, Victoria Berset, Lauren Moore

**Affiliations:** 1 School of Health sciences, Liverpool Hope University, Taggart Avenue, Liverpool L16 9JD, UK; 13002086@hope.ac.uk; 2 School of Agricultural, Forest and Food Sciences, Bern University of Applied Sciences, 3052 Bern, Switzerland; victoriaberset@hotmail.com

**Keywords:** non-nutritive sweeteners, stevia, glucose, appetite, food intake, diabetes, obesity

## Abstract

Non-nutritive sweeteners (NNS) are suggested to lower energy intake in the diet, but they have been paradoxically involved in the epidemic of obesity and Type 2 diabetes. Stevia is the least studied sweetener. This study aims to investigate the effect of stevia on postprandial glucose levels, appetite and food intake. Methods: 30 participants (20 females/10 males; 26.1 (10.56) years; body mass index (BMI) 23.44 (3.42) Kg/m^2^) took part in a three-arm crossover trial where they received preloads of water, sugar (60 g) and stevia (1 g) on three different days, followed by an ad libitum pizza lunch. Breakfast was standardised. A one-day diet diary was collected on each test day. Visual analogue scales (VAS) were used to assess subjective feelings of appetite. Blood glucose samples were collected at 30-min intervals until 120 min post lunch. Results: Energy intake did not significantly differ between preloads for ad libitum meals (*p* = 0.78) and overall day (*p* = 0.33). VAS scores for hunger and desire to eat (DTE) were lower following stevia preload compared to water (*p* < 0.05). After adjusting for the sugar preload and calorie content, postprandial glucose levels did not significantly differ between interventions. Conclusion: Stevia lowers appetite sensation and does not further increase food intake and postprandial glucose levels. It could be a useful strategy in obesity and diabetes prevention and management.

## 1. Introduction

Non-nutritive sweeteners (NNS) are sugar substitutes that have increased in popularity over the past two decades. The interest in NNS reside in their strong sweetening effects, without the further addition of sugar or energy to the diet. NNS include aspartame, saccharin, sucralose, stevia, cyclamate and acesulfame K [1].

NNS have been increasingly consumed as a means to lower energy intake [2] and therefore tackle the obesity and Type 2 diabetes epidemic; the latter currently accounts for 451 million cases worldwide. The continuous increase in the prevalence of Type 2 diabetes [3], along with its micro and macrovascular complications [4], constitutes a major burden on the health system. Postprandial glycaemia is an important predictor of diabetes risk and is suggested to precede the onset of fasting hyperglycaemia [5]. It is also strongly associated with diabetes complications, including cardiovascular diseases [6]. Therefore, approaches to lower postprandial glycaemia could have significant effects on diabetes prevention and management.

Despite providing minimal energy, NNS have been paradoxically involved in weight gain and Type 2 diabetes risk [7] through several mechanisms, including (i) increase in appetite and energy intake, (ii) disruption in the association between sweetness and calories, (iii) energy compensation following the intake of NNS, (iv) change in taste preferences and (v) alterations in gut microbiota [8]. Most of these effects have been identified in either animal or observational human studies [2]. Even though the interest in research on sweeteners has increased, there does not seem to be a current recommendation for NNS in relation to weight control and glucose management [9], which has left the public indecisive on whether the consumption of NNS is detrimental or beneficial to health. This is mainly due to the mixed results, the heterogeneity of the studies, the difference in study design and quality and the resultant complexity in drawing appropriate conclusions. The difficulty also relies on the significant difference in the chemical structure between NNS. Although they all have the ability to activate some taste receptors [10], NNS possess a different metabolic profile and can potentially exert varied effects on gut microbiota [7]. This affects the reliability of extrapolating the outcomes of one non-nutritive sweetener to another.

Stevia extract is a natural sweetener commonly referred to as stevia, and is obtained from the leaves of the Stevia plant. It is native to South America and has been used as a sweetener by the indigenous people for over 100 years [11]. Research on stevia has been limited and controversial.

While some studies showed a beneficial effect of stevia on improving glucose tolerance [12] and lowering postprandial glucose levels [13], others reported a larger increase in postprandial glucose levels after stevia consumption, compared to sugar [14]. Furthermore, stevia did not significantly affect self-reported satiety levels and food intake in one study [13], whereas an increase in appetite and food consumption has been reported by Tey et al. (2016) [14]. Most studies were, nevertheless, limited by a lack of a control group, as they compared stevia to sugar. The aim of this study was therefore to investigate whether stevia leads to an increase in glucose levels, appetite and/or food intake, when compared to water and sugar.

## 2. Materials and Methods

### 2.1. Participants

Participants were recruited through university email and word of mouth. Inclusion criteria included males and females, 18–65 years, body mass index (BMI): 18.5–29.9 Kg/m^2^. Exclusion criteria included history of diabetes or other chronic disease, allergies to stevia or the test meal and a diagnosed eating disorder. All subjects gave their informed consent for inclusion before they participated in the study. The study was conducted in accordance with the Declaration of Helsinki (2013), and the Ethics Committee of Liverpool Hope University approved the protocol.

### 2.2. Intervention

**Figure 1:**
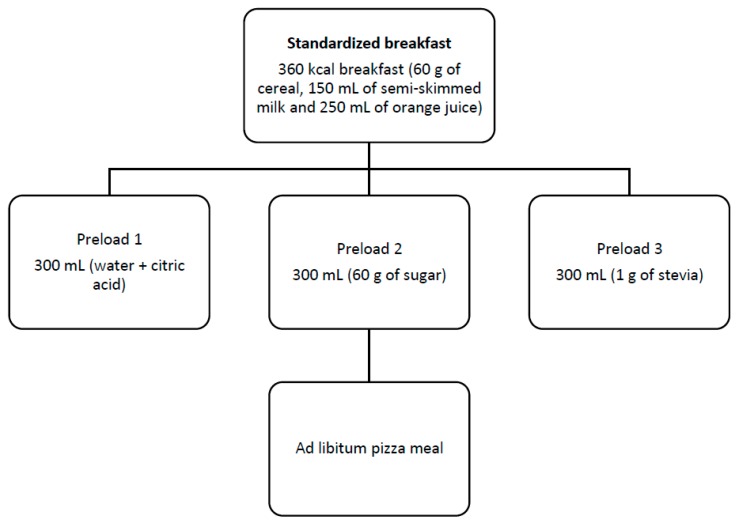
Study design.

**Figure 2:**
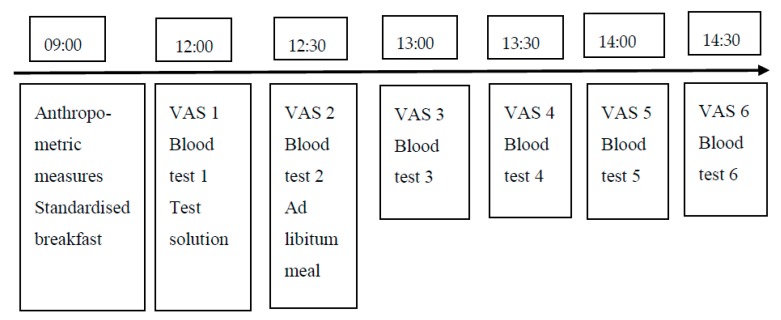
Timeline for each test day. VAS: Visual analogue scale.

The study was a three-arm single-blinded randomised crossover trial where participants received one of three different preloads (300 mL) containing (a) water mixed with small amounts of citric acid, (b) sugar (60 g) and (c) stevia (1 g) on three different days, and separated by a 4–5 day washout period. The quantity of sugar was selected to match the amounts commonly used in commercial sugary beverages. As for stevia, 1 g of this sweetener has been linked to a decrease in fasting blood glucose levels in the study of Ritu (2016) [15]; we therefore aimed to study how this dose affects postprandial glucose levels. The order of preloads was balanced in participants. On each test day, they were asked to attend the Lab at 9 a.m. after an 8-h fast. Anthropometric measures were taken and a general questionnaire was filled out only during the first visit. Participants then received a 360-kcal breakfast consisting of 60 g of cereals, 150 mL of semi-skimmed milk or unsweetened soy milk and 250 mL of orange juice. Three hours later, they received one of the three different preloads, followed by an ad libitum pizza lunch after 30 min (Figure 1). Pizzas and leftovers were weighed before and after consumption, and energy intake for each meal was calculated. A one-day diet diary was collected three times on each study day. The timeline for each intervention day is summarised in Figure 2.

Volunteers were asked to rate their hunger, desire to eat (DTE), fullness and satisfaction on 100 mm Visual Analogue Scales (VAS) with words anchored at each end, expressing the most positive and negative rating over a 180-min period before and after lunch, and every 30 min throughout the afternoon until 120 min post lunch.

Blood glucose samples were collected before preload and lunch, and then at 30-min intervals until 120 min after lunch. The area under the curve (AUC) for glucose was calculated. Blood samples were obtained by finger prick tests (Biosen C-Line) (Figure 2).

### 2.3. Anthropometric Measures

Height was measured using a stadiometer. The participant stood barefoot, with minimal clothes on, in a straight position, and the palms facing the thighs so that the posture was clear. The head was aligned according to the Frankfort plane.

Weight was measured in the morning at fasting using an electronic scale (Tanita BF-533, Body Fat Monitor/Scale). The participant wore light clothing and the scale was positioned on a flat surface.

Waist circumference was measured via a metal measuring tape. The tape was placed around the waist at the middle point between the lowest rib and the top of the hip bone, based on the protocol described by WHO (2008) [16].

### 2.4. Sample Size and Statistical Analysis

The determination of sample size was based on its ability to have 90% power to detect a clinically significant difference of 30% in AUC for glucose between interventions, with an alpha error of 0.05. Considering 20% attrition, 30 participants were recruited.

Continuous, normally distributed data were expressed as mean ± SD. VAS, AUC for glucose, food, energy and macronutrient intakes were analysed using one-way repeated measures ANOVA (Analysis of variance). Values for VAS and postprandial glucose levels were adjusted for baseline. For significant differences, changes over time were assessed via pairwise comparisons using the Bonferroni test. Diet diaries were analysed using Microdiet (v.3; v.4). Analysis was repeated with weight status (normal weight versus overweight) used as covariate. Significant changes were set at *p* ≤ 0.05.

## 3. Results

Thirty participants completed the study. The characteristics of the population are summarised in Table 1. The population was Caucasian and one participant had mixed ethnicity. Twelve participants were of normal weight (BMI between 18.5 and 24.9 Kg/m^2^) and nine were overweight (BMI >25 Kg/m^2^).

### 3.1. AUC for Glucose and Postprandial Glucose Levels

The analysis showed a significant effect of intervention (water, sugar and stevia) on AUC for glucose (F (2, 58) 11.83, *p* < 0.0001). Sugar preload resulted in a higher AUC for glucose compared to water *(p* = 0.001) and stevia (*p* = 0.007), while no significant difference between water and stevia preloads was noted (*p* = 0.2).

Postprandial glucose levels were significantly higher after sugar preload (*p* < 0.05). However, after adjusting for blood glucose values following preload, the difference was no longer significant.

### 3.2. Ad Libitum Lunch

**Figure 3:**
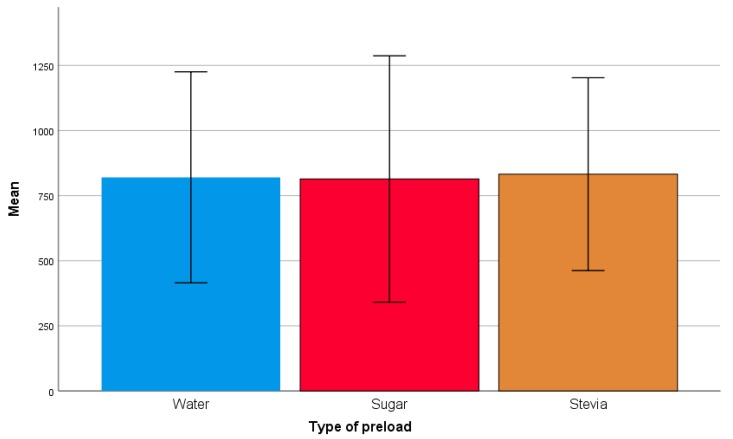
Energy intake from ad libitum meal following water, sugar and stevia preload consumption. *p* > 0.05.

Despite the difference in energy content between preloads, there was no significant effect of intervention on energy intake at lunch (F (2, 56) = 0.25, *p* = 0.78) (Figure 3).

### 3.3. Daily Energy Intake during Each Test Day

There were no significant differences in daily energy intake between water, sugar and stevia interventions (F (1.59, 44.59), *p* = 0.33). Participants did not compensate by consuming more energy during the day after the stevia preload (1660 ± 584 Kcal) compared to sugar preload (1771 ± 763 Kcal, *p* = 0.82) (Table 2).

### 3.4. Visual Analogue Scales

**Figure 4:**
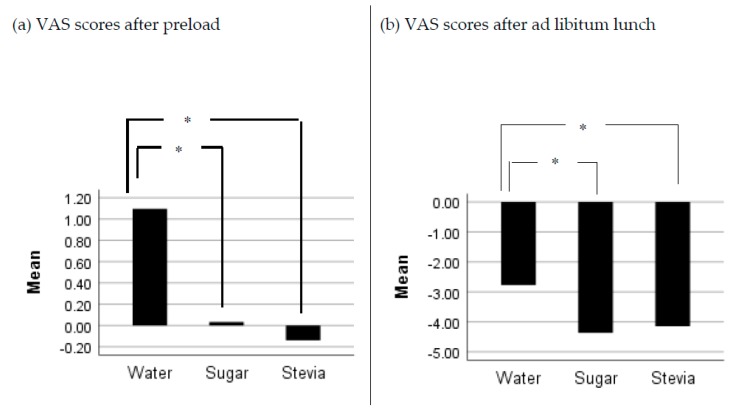
Hunger scores following preloads and ad libitum lunch. * *p* < 0.05.

There were no significant differences in reported scores of satisfaction and fullness between preloads after adjusting values for baseline (VAS1) (p > 0.05). However, there was a significant effect of preload on scores of hunger 30 min after preload (F (1.6, 45.2) = 4.35, p = 0.027). Participants scored higher rates of hunger following the intake of water preload compared to sugar and stevia preloads (p < 0.05), while no significant differences were noted between sugar and stevia. Similar results were reported in the VAS scores for hunger following lunch (F (2, 58) = 5.82, p = 0.05). Stevia resulted in lower subjective feelings of hunger compared to water (p = 0.039), while no significant differences between sugar and stevia were noted (p > 0.05) (Figure 4).

There was a significant effect of preload on DTE after preload intake (F (2.58) = 14.15, p < 0.0001). Participants scored a higher desire to eat following water intake (p = 0.001) compared to stevia and sugar intake, while there were no significant differences in ratings between sugar and stevia.

### 3.5. Effect of Weight Status on Response to NNS

A subgroup analysis based on BMI status (normal weight versus overweight) showed no significant differences between groups for VAS scores for fullness, hunger, satisfaction and desire to eat between groups. There were also no significant differences in energy intake at lunchtime (F(2, 54) = 1.41, *p* = 0.25)) or during the day (F(1.6, 43.4) = 1.06, *p* = 0.35)). Similar outcomes were noted for AUC levels for glucose (F (2, 56) = 1.52, *p* = 0.23)).

## 4. Discussion

This study aimed to assess whether stevia increased appetite and food intake compared to sugar and water, and leads to higher postprandial glucose levels following a meal. In our study, the higher calorie content of the sugar preload (240 Kcal) compared to water and stevia (virtually no calories) did not lead to a significant difference in energy intake at lunch, or during the day between preloads. These results are in line with the study of Anton et al. (2010) [13], which reported that stevia did not result in short-term compensation of food at lunchtime or during the day, when compared to sugar. Tey et al. (2016) [14] reported similar results. However, whether the compensation occurs over the long term remains to be investigated.

Compared to water, stevia led to lower subjective feelings of hunger and DTE after preload, and lower VAS of hunger before lunch (*p* < 0.05), with no resultant significant differences in energy intake. Interestingly, sugar and stevia resulted in similar satiety ratings compared to water. These outcomes are novel and have not been reported before. They could suggest that stevia has the potential to reduce appetite and consequently energy intake, yet the consumption of food in a laboratory setting might have affected the outcomes. Further research looking at the satiety effects of stevia compared to water and sugar need to be considered.

AUC for glucose was significantly higher after the sugar preload compared to water and stevia. This could solely be due to the caloric content of sugar. In fact, when we corrected for glucose levels after preloads, there were no significant differences in postprandial glucose levels (after the ad libitum meal) between the three preloads. This finding does not match with the study of Anton et al. (2010) [13], which noted a potential role of stevia in lowering postprandial glucose levels and managing postprandial hyperglycaemia. Furthermore, these results do not support in vitro and animal studies, which showed that stevia extract enhances insulin secretion and glucose absorption [17,18]. Long-term human intervention studies using stevia doses within the acceptable daily intakes (as set up by the European Food Safety Authority (EFSA), could help elucidate these effects.

Our findings suggest that stevia has at least a neutral effect on short-term food intake (it did not increase food palatability) and its consumption led to lower postprandial glucose levels compared to sucrose, providing more evidence that the link between Type 2 diabetes, obesity and the consumption of NNS is due to reverse causality.

Outcomes did not show significant effects of weight status (normal weight versus overweight) on the different outcomes. This might be due to the fact that our study was not powered enough to detect significant differences based on weight status. Further studies solely focused on the overweight and obese population need to be considered.

Our study has several limitations. In addition to the inclusion of free-living individuals, the study took place in a laboratory setting, which could have affected participants’ usual eating patterns. Our study was also single-blinded; while this is an advantage over open-label studies, participants were not aware of the preload content, which might have affected energy compensation after lunch or during the day. However, the strengths of the study include the presence of a control group (water) and the measurement of glucose and satiety at several intervals during the study.

## 5. Conclusions

Stevia intake did not lead to energy compensation during lunch or during the day, and resulted in lower postprandial glucose levels compared to sugar. Further studies looking at how stevia (in both food and drink) affects postprandial glycaemia and taste preferences are needed. Moreover, research looking at the long-term effects of stevia on glucose homeostasis and weight regulation in both normal weight and overweight people could help public recommendations, incorporate stevia into an overall healthy dietary pattern and reduce the intake of free sugars and energy intake. However, it is important to bear in mind that stevia, similar to other NNS, does not make the diet healthier; it makes it less unhealthy.

## Figures and Tables

**Table 1:** Characteristics of the studied population.

**Age (years)**	26.1 (10.56)
**Gender (M/F)**	10/20
**BMI (Kg/m^2^)**	23.44 (3.42)
**Waist circumference (cm)**	75.22 (8.77)

Age, body mass index (BMI) and waist circumference are expressed as mean (standard deviation).

**Table 2:** Daily energy and macronutrient intake during the three test meal days.

	Daily Energy Intake (Kcal)	Carbohydrates (g)	Protein (g)	Fat (g)
Water	1564 (981)	225.14 (124.38)	62.64 (41.67)	51.1 (43.1)
Sugar	1771 (763)	251.64 (122.66)	69.37 (39.8)	53.29 (27.7)
Stevia	1660 (584)	223.30 (87.67)	66.7 (30.42)	57.51 (22.44)

Differences in energy and macronutrient intake were not significant between groups (p>0.05).
